# Radioembolization in the Treatment of Neuroendocrine Tumor Metastases to the Liver

**DOI:** 10.4061/2011/785315

**Published:** 2011-12-22

**Authors:** Martin Vyleta, Douglas Coldwell

**Affiliations:** Department of Radiology, University of Louisville School of Medicine, Louisville, KY 40202, USA

## Abstract

Surgical excision remains the preferred treatment for resectable hepatic metastases of neuroendocrine tumors. In cases of more disseminated hepatic disease, transarterial radioembolization with Yttrium-90- (90Y-) labeled microspheres has been demonstrated as a viable option for symptom and locoregional tumor control. On an outpatient basis, radioembolization can be utilized from early line to salvage phases, in various combinations with systemic therapies. Review of available data shows encouraging safety and efficacy profiles for the intraarterial application of 90Y for the treatment of mNETs of the liver. Symptom control and decrease in somatostatin analog use can be achieved, as well as prolonged survival.

## 1. Introduction

After removal of the primary, isolated metastases of neuroendocrine tumors to the liver can be curatively approached by surgical resection with achievable disease-free survival of 42 to 46 months depending on the extent of metastases [1]. However, only 10% of metastatic liver disease cases are resectable [2]. Also, significant surgical morbidity and mortality as well as the unpredictable natural history of malignant neuroendocrine neoplasms has to be considered [2]. Consensus guidelines for digestive (neuro)endocrine tumors currently call for complete hepatic deposit resection or 90% debulking if feasible [3] since their introduction in the 1980s somatostatin analogs have significantly helped in symptom control of functional neuroendocrine tumors. Their effectiveness, however, tends to diminish over time due to tachyphylaxis and disease progression. An antiproliferative effect has been ascribed to somatostatin analogs on their own [4]. Additional labeling with radionuclides (e.g., 177Lutetium-DOTA-TATE) has shown response rates of 13–30% and median overall survival of 13–46 months along with significant clinical benefit [5–10].

Overall, metastatic neuroendocrine tumors have exhibited modest response to systemic chemotherapy. Streptozocin- and doxorubicin-based protocols achieve response rates of up to 16%. In more poorly differentiated NETs a 41.5% response rate has been seen with cisplatin/etoposide combinations [6–10]. Recently, 70% partial remissions with estimated overall survival of 92% at 2 years have been reported with a temozolamide/capecitabine regimen in metastatic NET of the pancreas [11].

Novel agents targeting diverse receptors are in various stages of development. Preliminary data for sunitinib, a multikinase inhibitor, for example, demonstrate a promising progression-free survival of 11.4 months versus 5.5 months for placebo.

## 2. Transarterial (CHEMO) Embolization (TA[C]E)

Retrospective studies have shown benefit to both TAE and TACE in the treatment of mNET with regard to tumor growth, symptom control, and biochemical surrogates. In the most recent large retrospective study of 123 treated patients undergoing an average of 7 chemoembolization cycles each, 62% partial response was seen with overall 3-, 5-, and 10-year survivals of 59, 36, and 20%, respectively, and overall mean survival of 5.47 years [12].

 This result is corroborated by the 67% ORR (mean survival 33.8 months) in another large study employing TACE and TAE for the treatment of carcinoid hepatic metastases. A separate group of islet cell carcinomas in the latter study exhibited an ORR of only 34% (mean survival 23.2 months); in the former study 10 cases with insulin/glucagon secretion were subsumed in the study population [12, 13].

No consensus has been established regarding technique, embolizing, and chemotherapeutic agents in TACE. Overall, both TAE and TACE appear to elicit similar responses from NET liver metastases, suggesting relative primacy of the embolic effect [2, 14].

## 3. Transarterial Radiombolization (TARE)

Transarterial radioembolization (TARE) or selective internal radiotherapy (SIRT) with Yttrium-90 microspheres represents a further viable option in the treatment arsenal for nonresectable liver metastases of both functional and nonfunctional NETs. A number of retrospective as well as a few prospective studies demonstrate efficacy and safety combined with the convenience of an outpatient procedure that rarely requires hospitalization (Table 1) [15–25]. What is more, it appears that the primarily one-time treatments with TARE compare favorably to usually multiple procedures required TA(C)E regimens.

Two kinds of Yttrium-90 microspheres are currently approved for treatment of unresectable liver tumors on the European market: Yttrium-90 resin microspheres (SIR-spheres; Sirtex Medical, Sydney) with 20–60 *μ*m diameter mounted with a radioactive load of approximately 50 Bq per sphere, as well as 20–30 *μ*m glass microspheres (TheraSphere, MDS Nordion, Ottawa) with the higher radioactive load of 2500 Bq. SIR-spheres come with premarket approval (PMA) for unresectable hepatic metastases of colorectal cancer in the United States and may be utilized “off-label” for other tumors without prejudice under the using physicians discretion authority and responsibility. Therasphere has a humanitarian device exception for the treatment of hepatocellular carcinoma in the US which is more restrictive than PMA and requires an IRB protocol and limitations to hepatocellular carcinoma (HCC) only.

A phase II trial of Yttrium-90 resin microspheres in combination with systemic FU chemotherapy was conducted with 34 patients. While this represented first-line therapy for the majority of patients, 29% had failed prior liver resection and 15% had received earlier chemotherapy, with 59% also manifesting extrahepatic disease. Complete response was effected in 18%, partial response in 32%, and stable disease 15% of cases. Symptom improvement and Chromogranin A decrease at 6 months were seen in 50% and 41% of cases, respectively. 59% of patients remained alive at 37 ± 2 months, with 12 patients without hepatic recurrence at 33.3 ± 2.3 months [20].

Another prospective trial with Yttrium-90 resin microspheres involved ten patients with progressive or symptomatic unresectable hepatic spread of NET. 40% partial response was seen at 6 months and 2 out of 3 patients experienced symptomatic improvement, and average quality of life climbed to general-population levels by 6 months. Seven patients were still alive at 35 months followup, with the three intervening deaths attributable to progression of extrahepatic disease. Little toxicity was observed, with the authors noting comparable to better toleration of TARE compared to TACE [23].

Another study with Yttrium-90 resin microspheres also had quality of life as an endpoint and could demonstrate significant improvement of quality of life [18].

In a third prospective trial, 20 and 22 patients with mNET were treated with resin and glass Yttrium-90 microspheres, respectively, after the patient had previously undergone surgery (36%), ablation (19%), TA(C)E (14%), and symptomatic treatment (60%). ORR resulted in 50% and 54%, and stable disease in 44% and 38.5%, respectively. Projected median survival times were 28 and 22 months, respectively (not statistically significant difference), with the majority of patients alive at time of study publication [22]. 

The largest retrospective study to date reviewed a total of 148 patients in 10 clinical centers treated with Yttrium-90 resin microspheres in a primarily salvage setting of mNET. 70 months median survival with 63% ORR and 23% stable disease were demonstrated, with most deaths due to progression of extrahepatic disease. Toxicity was very low, no radiation-induced liver disease (RILD) was seen even in the 33 patients receiving retreatment of the same liver lobe(s) [19].

Repeated radioembolizations of both or single lobes were also performed in a minority of patients in other studies, with a few receiving a third treatment [19, 20, 24, 25]. No case of RILD was seen and an increased duration of tumor response in cases of hepatic mNET progression was recognized [18, 19, 24, 25].

## 4. Discussion

Overall, the available studies demonstrate effective and safe use of radioembolization for liver dominant disease in mNET. There is a positive effect on symptoms, quality of life, response criteria, and survival in various stages of progression, which is mirrored in the recommendations of the Radioembolization Brachytherapy Oncology Consortium [26]. A robust safety profile for radioembolization with Yttrium-90 microspheres was confirmed in two 2009 analyses [26, 27]. The overall incidence of RILD, in particular, was estimated to be 0.8% [26].

Evidence-based data for treatment decisions in inoperable mNET are not presently available. A recent overview of strategies for advanced enteropancreatic NET comprehensively presents the panorama of current and developing multimodal treatment options. In general, former wait-andsee strategies even in well-differentiated tumors with minor-to-moderate tumor loads will have to be reevaluated, as tumor progression, with median TTP of 6 months, is inevitable [5]. 

In order to relieve the common carcinoid syndrome consisting of diarrhea, flushing, hypertension, bronchoconstriction, right valvular heart failure and other endocrine effects, or effect medication sparing, at least palliative tumor debulking, either surgically or with transarterial approaches, is necessary. Hepatic tumor burden debulking appears to improve survival as well [28–32]. 5-year survival rates for mNET are 50–59% according to national cancer registries [33, 34]. With intervention, 5-year survival reaches 72–100% in limited hepatic spread and 25–51% in more extensive hepatic spread [35, 36].

TARE (see Table 1) is at least comparable to TA(C)E in effectiveness [13, 14]. An advantage of the TARE protocol is the generally single treatment to achieve this effect, while TA(C)E is generally applied multiple times. This obvious patient comfort and quality of life advantage is compounded by the well-known lower severity of the “postembolization” syndrome (which in TARE's case represents radiation irritation) and reduced need for inpatient admittance. On the other hand, preparatory angiography mapping, embolization, and treatment simulation are a necessary first step in TARE workup.

It is conceivable that Yttrium-90 microspheres can be held in reserve, so to speak, for further repeated use to establish prolonged disease control. In this respect, it seems superior to repeated TA(C)E. For one thing further extended use of TA(C)E is limited by the sheer number of necessary repetitions, reducing patient days outside the hospital and thus quality of life. The extended use of TARE, on the other hand, would likely not reach the number of regular TA(C)E sessions. Similar advantages also apply to side effects and outpatient flexibility. Also, the degree of embolic effect could skew repeated treatment schemes to TARE's favor: while TA(C)E may “prune” the arterial tree to such an extent that agent delivery is impaired due to the larger particle size (100–300 *μ*m versus 35 *μ*m for TARE), the lesser embolic effect of TARE may keep the path to the tumor more reliably open. Further examination of the limits of dose and repeat application with regard to RILD could possibly enable attempts at treating even more advanced hepatic spread with radioembolization. An investigation of radiosensitizers, for example, capecitabine, to be concurrently used with TARE may also be warranted as this could represent another lever to further potentiate its effects [37].

Apparently disease control through radioembolization treatment is effective and long-lasting enough for most patients in the observed time spans to succumb to progressive extrahepatic disease. Thus, initiation/resumption of chemotherapy, possibly combined with extrahepatic local resection/ablation if necessary, appears reasonable.

Further considerations concern the endpoints surveyed in Yttrium-90 treatment studies. Morphologic response criteria (i.e., RECIST) may be misleading in TARE followups. Since the RECIST criteria were designed to reflect the response to systemic chemotherapy, the response to TARE is not gradual shrinkage but the creation of a necrotic tumor whose margins may not be well circumscribed in the baseline CT yielding a reading of progressive disease or mere stable disease. Collection of tumor marker data, for example, Chromogranin A, as well as functional parameters, for example, PET, may help to overcome some of these shortcomings. Emphasis should also be laid on formal measurements of quality of life in subsequent studies of TARE to better gauge this essential patient dimension.

## Figures and Tables

**Table 1:** Study outcomes of TARE for NET liver metastases.

Lead Author, Year	*n*	Prospective?	Treatment Phase	ORR	SD	Symptom Response	Survival
Arslan, 2011 [17]	10	No	?	80%	10%	NR	All alive at 4–28 mo
Cao, 2010 [15]	51	No	?	38%	27%	NR	36 mo
Saxena, 2010 [26]	48	No	?	55%	23%	NR	35 mo
Kalinowski, 2009 [19]	9	Yes	Refractory	66%	33%	Improved QoL	57% alive at 36 mo
Kennedy, 2009 [27]	148	No	Refractory/salvage	63.2%	22.7%	NR	70 mo
King, 2008 [22]	34	Yes	First-line*	50%	14.7%	50%	59% alive at 37 ± 2 mo
Murthy, 2008 [16]	8	No	Refractory/salvage	12.5%	50%	NR	14 mo
Rhee, 2008 [23]	20 22**	Yes Yes	Refractory Refractory	50% 53.8%	44% 38.5%	NR NR	28 mo 22 mo
Meranze, 2007 [24]	10	Yes	First-line	40%	60%	Improved QoL	70% alive at 35 mo
McGrath, 2007 [25]	26	No	First-line/refractory	58.3%	33%	NR	69% alive at 17.3 mo
Kennedy, 2006 [21]	18	No	First-line/refractory	89%	NR	NR	89% alive at 27 mo

*combined with 5-FU.

**with Yttrium-90 glass microspheres, all others with Yttrium-90 resin microspheres. Median survival in last column unless otherwise indicated. ORR: objective response rate (complete + partial response). SD: stable disease. NR: not reported. QoL: quality of life.
